# A Case Report Evaluating Gastric Emphysema versus Emphysematous Gastritis

**DOI:** 10.21980/J8ZH26

**Published:** 2024-04-30

**Authors:** Anna Nguyen, Mark Slader, Lindsey Spiegelman

**Affiliations:** * California University of Science and Medicine, Colton, CA; ^ University of California, Irvine, Department of Emergency Medicine, Orange, CA

## Abstract

Gastric emphysema (GE) and emphysematous gastritis (EG) share similar clinical presentations but exhibit drastically different prognoses. While GE is generally benign, EG is associated with mortality rates up to 60%. Here, we present the case of a 29-year-old female patient who presented to the emergency department (ED) with symptoms of nausea, vomiting, and epigastric abdominal pain. Clinical evaluation revealed tachycardia, pain out of proportion, leukocytosis, and metabolic acidosis. Computed tomography (CT) scan unveiled the presence of air within the gastric wall, and a presumptive diagnosis of gastric emphysema was made. The patient responded positively to conservative management and was discharged after a two-day hospitalization. This case report emphasizes the need for physicians to adeptly distinguish between GE and EG. Timely identification and precise differentiation of the two conditions allow for timely and tailored management, ultimately leading to improved clinical outcomes in patients. By providing insights into the etiologies, clinical presentations, and imaging findings for the two pathologies, we aim to empower clinicians to make informed decisions for optimal patient care.

**Topics:**

Gastric emphysema, emphysematous gastritis, gastric pneumatosis.

[Fig f1-jetem-9-2-v10]

**Figure f1-jetem-9-2-v10:**
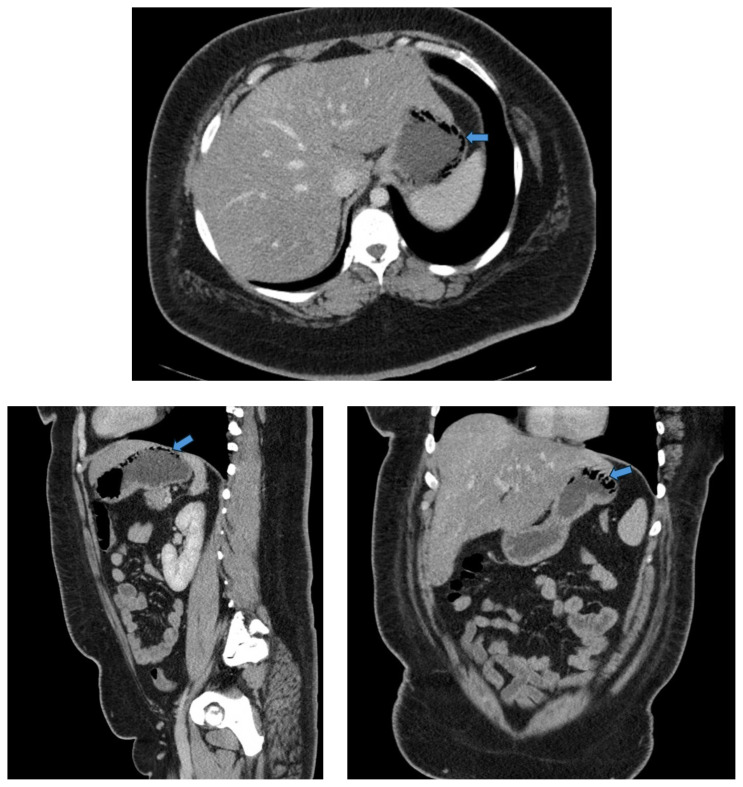
CT Video Link: https://youtu.be/zMVLLRbTB0w

## Brief introduction

Gastric pneumatosis refers to a rare condition in which air is present within the stomach wall. It can be further classified into two categories: gastric emphysema (GE) and emphysematous gastritis (EG). While the two pathologies can present similarly clinically and on imaging, gastric emphysema is usually self-limiting with benign outcomes whereas emphysematous gastritis is more commonly associated with a poor prognosis and significantly higher mortality rates, ie, 53.3% in EG vs 29.4% in GE.^1^ As such, it is important to highlight the distinctions between the two conditions to allow for rapid diagnosis and effective clinical management.

Gastric emphysema and emphysematous gastritis differ primarily in their etiology; most cases of GE are non-infectious in nature while EG is often caused by infectious agents.^1^, ^5^ The pathogenesis of these conditions is not yet fully understood, but there are multiple theories that have been inferred with evidence from prior cases. Because there are numerous ways in which air can enter the stomach wall, there exists many etiologies that can lead to gastric emphysema. These include, but are not limited to, mechanical injury to the mucosa, gastric outlet obstruction (secondary to pyloric stenosis, carcinoma, etc.), and severe vomiting.^3^,^4^ In contrast, emphysematous gastritis is typically the result of a gas-producing pathogen and has been linked to a range of microorganisms, including gram-positive, gram-negative, anaerobic, and fungal organisms. Infection with these pathogens results in gas production within the gastric wall but can also lead to fatal necrosis of the stomach wall.^1^

The differences in outcome and mortality between gastric emphysema and emphysematous gastritis make it imperative that providers be able to distinguish between the two conditions. Here we present a case of a 29-year-old female diagnosed with gastric emphysema after a sudden onset of nausea, emesis, and abdominal pain. This paper will also further discuss the etiologies, risk factors, clinical findings, and most importantly, diagnostic and management procedures in both cases of gastric emphysema and emphysematous gastritis. New images are also included to help visualize expected findings. Patient-written consent for medical photography was obtained during the patient’s admission.

## Presenting concerns and clinical findings

A 29-year-old female with past medical history of gastroesophageal reflux disease and bipolar I disorder presented to the emergency department (ED) complaining of intractable nausea, vomiting, and diarrhea that started that morning. Symptoms of abdominal bloating and discomfort woke her from sleep and were followed by six to eight episodes of vomiting and diarrhea. She began to experience severe epigastric pain, prompting her visit to the ED. She denied fever, chest pain, shortness of breath, dysuria, hematuria, hematemesis, melena, or hematochezia. She further denied recent changes in her diet, sick contacts, and recent travel. She endorsed daily marijuana use and denied alcohol or tobacco use. She reported one similar episode two months prior that resolved with minimal intervention at an outside ED. In the ED, she was afebrile with a temperature of 97.3 °F, tachycardic to 133 beats per minute, blood pressure of 146/93 mmHg, respirations of 24 per minute, and pulse oximetry of 98% on room air.

## Significant findings

The patient’s physical exam revealed an uncomfortable-appearing female. Her abdomen was soft, non-distended, and non-tender with epigastric pain that was out of proportion to her exam. The remainder of her physical exam was unremarkable.

Key laboratory findings highlighted an elevated white blood cell count (WBC: 19.3 thous/mcl), concerning for possible intraabdominal infection and meeting sepsis criteria with concurrent tachycardia. Critical abnormalities in the patient’s metabolic panel included a metabolic anion gap acidosis with elevated anion gap (17 mmol/L) and decreased bicarbonate level (15 mmol/L). This was presumed to be secondary to lactic acidosis, although lactate levels were not initially measured. Her lipase level did not suggest pancreatitis.

For further evaluation, a CT scan of the abdomen and pelvis was obtained and revealed gas within the gastric wall at the fundus (blue arrows), concerning for gastric emphysema versus emphysematous gastritis. There was no gastric wall thickening, free air, bowel obstruction, drainable fluid collection, or evidence of portal venous gas. Incidentally, hepatomegaly and likely hepatic steatosis were also noted.

## Patient course

After initial evaluation, the patient was given intravenous fluids, analgesic medication, and antiemetics (ondansetron). Blood cultures were drawn and piperacillin-tazobactam was started once the CT abdomen and pelvis findings resulted. On reevaluation, the patient’s nausea, vomiting, and epigastric pain had resolved. However, given her imaging findings, emergency general surgery (EGS) and gastroenterology (GI) were consulted.

The EGS service evaluated and was reassured by the patient’s improved abdominal exam. They favored a diagnosis of gastric emphysema over emphysematous gastritis, and recommended medical management with bowel rest, maintenance intravenous fluids, proton pump inhibitor therapy, and agreed with broad spectrum antibiotic coverage. They performed serial abdominal exams and noted continued patient improvement. The GI service came to a similar conclusion, favoring a viral or bacterial gastroenteritis that induced significant vomiting and resulted in gastric emphysema. Neither service recommended surgical or procedural intervention.

The patient was admitted for further observation and was continued on empiric piperacillin-tazobactam coverage. Her leukocytosis resolved. She was initiated on a clear liquid diet and advanced to her normal diet without difficulty. Her infectious work up was unrevealing with negative blood cultures, urinalysis, and stool cultures. She was discharged the day after admission, referred to her primary care physician for further follow up, and given strict return precautions.

## Discussion

Gastric emphysema is a relatively rare condition, but because of its similarities to the much more lethal emphysematous gastritis, it is crucial for health care providers to be able to distinguish between the two conditions.^1^,^2^,^7^ There are several clinical conditions that may lead to gastric emphysema, which can be classified into three categories: traumatic, obstructive, and pulmonary. Traumatic gastric emphysema occurs when the stomach wall sustains physical damage, resulting in tears in the mucosa. This damage can be caused by procedures such as gastrostomies, endoscopies, or nasogastric tube placements.^8^ Obstructive gastric emphysema is caused by a distal obstruction to the stomach. The resulting increase in gastric distension can sometimes cause mucosal tears, allowing luminal air to diffuse through. Some causes of obstruction include peptic ulcer disease, carcinoma, duodenal obstruction, pyloric stenosis, or gastric volvulus.^2^,^8^ Lastly, the rarest type of gastric emphysema is the pulmonary type, where air from the mediastinum penetrates into the gastric wall due to increased intrapulmonary pressure.^2^,^8^ Several pathologies allow for this, including chronic obstructive lung disease, asthma, pneumothorax, alveolar rupture, etc.^2^,^4^,^8^,^9^ Outside of these categories, ischemia or infarction of the small bowel or colon can also sometimes allow for air to enter the gastric wall.^10^ Predisposing factors for gastric emphysema include gastric ischemia, atherosclerosis, hypertension, alcoholism, and diabetes mellitus.^3^

In emphysematous gastritis, the presence of air in the stomach wall is secondary to infection with gas-forming pathogens. The most common species involved in this infection are Klebsiella pneumoniae, Escherichia coli, Enterobacter spp., Pseudomonas aeruginosa, and Candida spp.^1^,^7^ Predisposing factors for emphysematous gastritis include ingestion of corrosive substances, alcoholism, recent abdominal surgery, and gastroenteritis.^7^

Gastric emphysema and emphysematous gastritis have similar clinical manifestations, with the latter usually having additional deteriorating findings. Commonly, both conditions present with nonspecific gastrointestinal symptoms like nausea, vomiting, abdominal pain, bloating, and indigestion.^9^,^11^ Hematemesis can occasionally occur due to mucosal tears.^3^ In patients with emphysematous gastritis, symptoms of acute infection such as fever or mental status change are often seen as well.^1^

Currently, computed tomography (CT) of the abdomen is the method of choice when evaluating for gastric emphysema or emphysematous gastritis. It is more sensitive and specific as compared to plain radiographs when evaluating for small amounts of air within the gastric wall.^9^ Additionally, CT allows for evaluation of other intraabdominal pathologies.^12^ Although both gastric emphysema and emphysematous gastritis can present similar radiographic features, there are a few differences that can aid in differentiation. Gastric emphysema typically presents as a hypodense linear or curve fringe on the gastric wall along with gastric distension and the absence of gastric wall thickening.^8^ This is in direct contrast to emphysematous gastritis which presents as a streaky distribution of air along with evidence of gastric wall thickening.^3^ It is also important to note that presence of extragastric air in other bowel segments is a strong indicator for emphysematous gastritis.^5^ In our patient’s case, the CT scan’s identification of air within the gastric wall without accompanying wall thickening or evidence of extragastric air favored a diagnosis of gastric emphysema rather than emphysematous gastritis.

The prognosis of gastric emphysema is generally benign with the chance of recurrence being extremely rare.^8^ Spontaneous resolution sometimes occurs even without a specific treatment.^9^ Hemodynamic status plays a key role in determining the management of gastric emphysema. In hemodynamically stable patients, conservative treatment is usually effective; this includes bowel rest, intravenous fluids, antiemetics, broad-spectrum antibiotics, and proton-pump inhibitors.^1^,^3^ If the patient is hemodynamically unstable with evidence of an acute abdomen, immediate surgical exploration may be indicated.^3^ In contrast, due to its poor prognosis, emphysematous gastritis often requires aggressive treatment. Management with fluids and broad-spectrum antibiotics early in the course of the disease has been noted to improve outcomes.^13^ For patients with evidence of transmural ischemia, perforation, uncontrolled sepsis, or other signs of clinical deterioration, surgical intervention with partial or total gastrectomy is then considered.^1^ There is, however, ongoing debate as to whether total gastrectomies should be performed because active infection can significantly delay or prevent the healing process.^4^

This case report highlights and reviews two conditions associated with the presence of gastric pneumatosis: gastric emphysema and emphysematous gastritis. Although these conditions share similar clinical presentations, their outcomes and mortality rates differ significantly. Gastric emphysema is typically self-limiting and benign, while emphysematous gastritis requires aggressive treatment and often results in poor outcomes despite major surgical interventions. It is therefore crucial for clinicians to be well-informed about both entities to provide timely diagnosis and efficient management.

## Supplementary Information








