# Supplementary figures and images for: A Case Report Evaluating Gastric Emphysema versus Emphysematous Gastritis
Source: J Educ Teach Emerg Med. 2024 Apr 30;9(2):V10–4. doi: 10.21980/J8ZH26 (PMC11068322; doi:10.21980/J8ZH26)

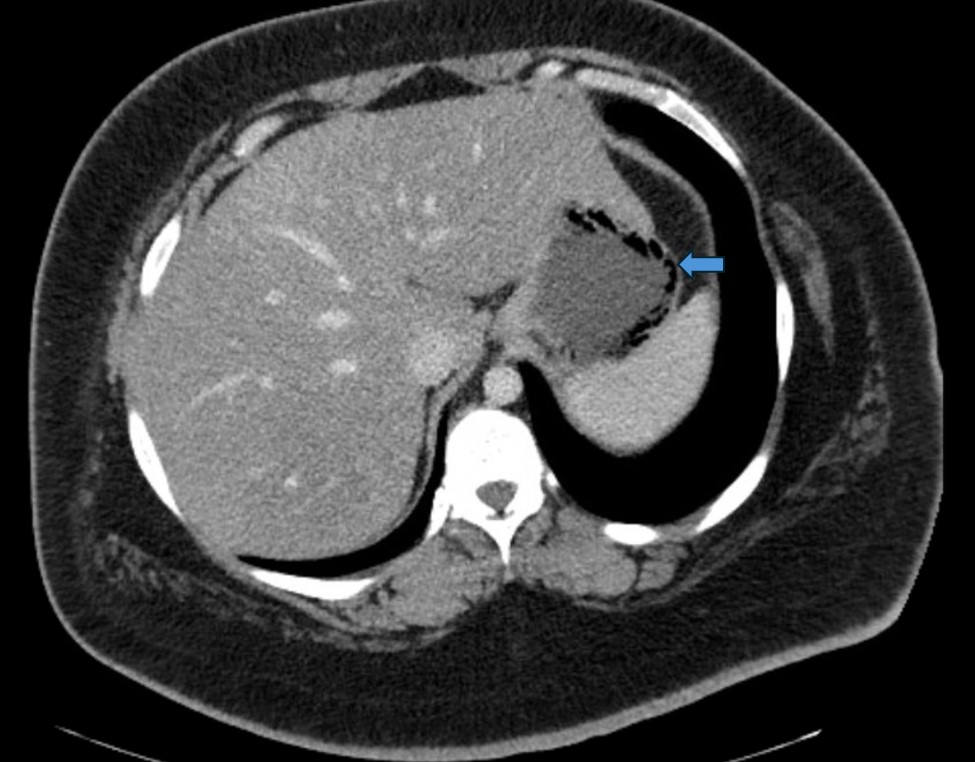

Supplement: Supplementary file 2 [file jetem-9-2-V10-supp2.jpg]

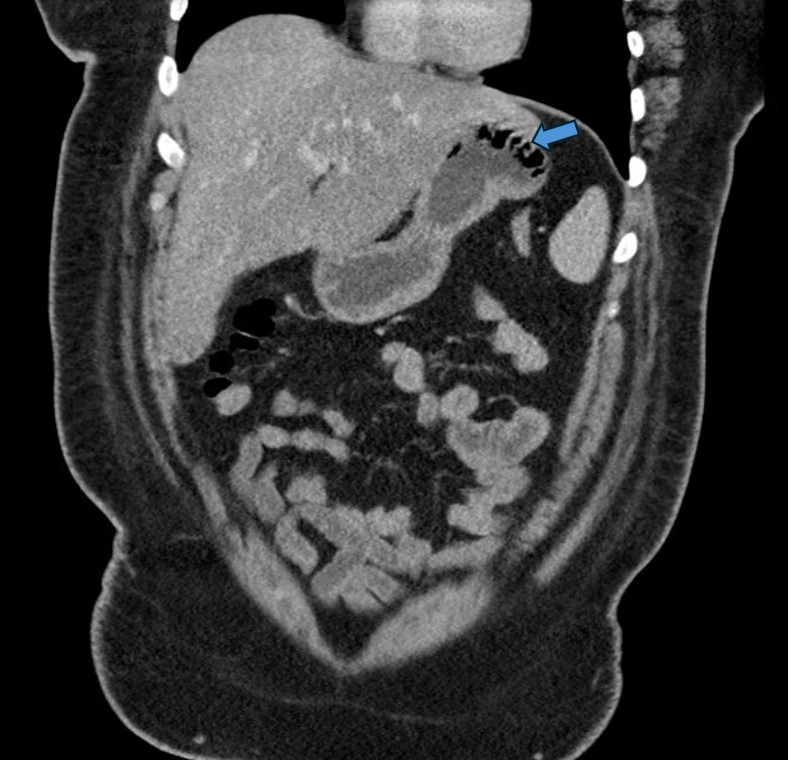

Supplement: Supplementary file 3 [file jetem-9-2-V10-supp3.jpg]

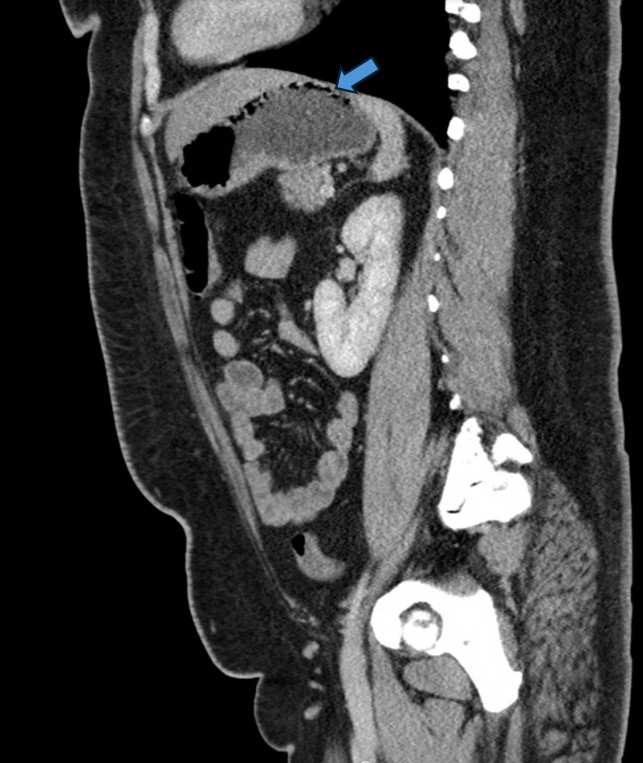

Supplement: Supplementary file 4 [file jetem-9-2-V10-supp4.jpg]
